# Baitouweng decoction alleviates ulcerative colitis by regulating tryptophan metabolism through DOPA decarboxylase promotion

**DOI:** 10.3389/fphar.2024.1423307

**Published:** 2024-06-21

**Authors:** Junzhi Zhang, Binyan Lin, Ying Zhang, Xiaochao Hu, Tongtong Liu, E-Hu Liu, Shijia Liu

**Affiliations:** ^1^ The Affiliated Hospital of Nanjing University of Chinese Medicine, Jiangsu Province Hospital of Chinese Medicine, Nanjing, China; ^2^ College of Pharmacy, Nanjing University of Chinese Medicine, Nanjing, China

**Keywords:** Baitouweng decoction, ulcerative colitis, dopa decarboxylase, tryptophan metabolism, AhR, intestinal barrier

## Abstract

**Background:**

Baitouweng decoction (BTW) is a classic botanical drugs formula that has been widely used clinically for the treatment of gut-related disorders in China. However, its role in ameliorating ulcerative colitis (UC) remains to be explored.

**Purpose:**

The study aimed to determine the therapeutic efficacy and potential mechanism of action of BTW on dextran sodium sulfate (DSS)-induced colitis mice.

**Methods:**

*In vivo*: 3.5% DSS-induced experimental colitis mice were treated with BTW (*Pulsatilla chinensis* (Bunge) Regel, *Phellodendron chinense* C. K. Schneid, *Coptis chinensis* Franch and *Fraxinus chinensis* Roxb), kynurenine or DOPA decarboxylase (DDC) inhibitor (carbidopa). *In vitro*: Caco-2 cells were stimulated with TNF-α to activate inflammation and later treated with various concentrations of BTW and carbidopa. Model evaluation included body weight, disease activity index (DAI) score, colon length and histopathology. Cytokine levels were measured by flow cytometry. Protein levels were analyzed by proteomics and functionally annotated. The levels of tryptophan metabolites in mouse serum and colon were detected by liquid chromatography-tandem mass spectrometry (LC-MS/MS). Alcian Blue/Phosphate Acid Schiff (AB/PAS) staining, immunohistochemistry and western blot were used to assess the intestinal barrier function and detect the protein expression levels.

**Results:**

BTW significantly reduced the DAI, ameliorated colonic injury and regulated inflammatory cytokines in DSS-induced colitis mice. The botanical drugs formula also promoted intestinal epithelial barrier repair by enhancing the expression of the tight junction (TJ) proteins. Tryptophan metabolic signaling pathway was significantly enriched in DSS-induced UC mice, and BTW decreased the level of kynurenine, increased indole metabolites. The therapeutic effect of BTW was evidently reduced when kynurenine was given to mice. Also, BTW promoted DDC protein expression and activated the aryl hydrocarbon receptor (AHR)/IL-22 signaling pathway.

**Conclusion:**

BTW improves ulcerative colitis by promoting DDC expression, regulating the conversion of tryptophan metabolism from the kynurenine pathway to the indole metabolism pathway, thereby modulating tryptophan metabolism to increase indole metabolites, and activating AHR receptors to restore intestinal barrier function.

## 1 Introduction

Ulcerative colitis (UC) is a lifelong inflammatory disease and the prevalence of UC was estimated to be 5 million cases worldwide in 2023 (Liu et al., 2022; Le Berre et al., 2023). The pathogenesis of UC is complex and incompletely understood, it is classically thought to be mediated by epithelial barrier defects, dysregulated immune responses and dysbiosis (Le Berre et al., 2020). The main clinical treatment for UC, such as salicylic acid, glucocorticoids, and immunosuppressants, is focused mainly on controlling intestinal inflammation. However, long-term or high-dose medications can induce drug resistance and cause various adverse effects, ultimately preventing the treatment of persistent UC (Lamb et al., 2019).

L-Tryptophan (Trp) is an essential amino acid for intestinal mucosal cells, and is involved in intestinal inflammation, the epithelial barrier and host energy homeostasis (Roager and Licht, 2018; Taleb, 2019). Trp metabolism is closely associated with both inflammation and cancer, and most of the dietary tryptophan is metabolized by three known pathways, including kynurenine pathway, indole metabolism pathway and serotonin metabolism pathway (Xue et al., 2023). Studies have shown that Trp metabolism through the kynurenine pathway is involved in the regulation of immunity, neuronal function and intestinal homeostasis (Shi et al., 2024). Approximately 1%–2% of Trp can be converted into serotonin in the enterochromaffin cells of the intestinal mucosa (Kałużna-Czaplińska et al., 2017). In addition to the kynurenine pathway and serotonin pathway, approximately 4%–6% of Trp is metabolized into indole compounds through indole metabolism pathway, including indole-3-pyruvate, tryptophol (Indole-3-ethanol, IET), indole-3-acetaldehyde, indole-3-acetic acid (I3A), indole-3-lactic acid and indole-3-propionic acid (Scott et al., 2020). These indole metabolites can bind to aryl hydrocarbon receptor (AHR) to promote intestinal homeostasis, enhance barrier function and tight junction (TJ) proteins, reduce colonic permeability (Taleb, 2019). Dopa decarboxylase (DDC) is an important metabolic enzyme in Trp metabolism, which serves as an emerging biomarker for Parkinson’s disease in cerebrospinal fluid. DDC metabolizes tryptophan to tryptamine, while IET and I3A are downstream metabolites of tryptamine (Maini Rekdal et al., 2019).

Baitouweng decoction (BTW), classic botanical drugs formula, was first described in the “Treatise on Febrile Diseases” written by the renowned Chinese physician ZhongJing Zhang. BTW contains four traditional botanical drugs, *Pulsatilla chinensis* (Bunge) Regel, *Phellodendron chinense* C. K. Schneid, *Coptis chinensis* Franch and *Fraxinus chinensis* Roxb, which have been used for centuries to treat intestinal diseases such as UC. According to preclinical studies, BTW has a variety of pharmacological effects including hepatoprotective, anti-inflammatory, antimicrobial, antitumor and antioxidant effects (Zhang et al., 2009). BTW also restores the damage of intestinal barrier and intestinal mucosa in DSS-induced UC (Pan et al., 2024), however, the underlying mechanism remains to be explored.

In this study, we first evaluated the therapeutic effect of BTW in DSS-induced UC mice. Next, using proteomics, we predicted the possible targets and pathways for the treatment of UC in BTW. Metabolic changes associated with UC were illustrated by Targeted metabolite analysis. Also, we verified that BTW affects tryptophan metabolites through increasing DDC expression, which activates the AHR/IL-22 pathway and improves the intestinal barrier.

## 2 Materials and methods

### 2.1 Chemicals and reagents

BTW is composed of *P. chinensis* (Bunge) Regel (15 g), *P. chinense* C. K. Schneid (12 g), *C. chinensis* Franch (6 g) and *F. chinensis* Roxb (12 g). *Pulsatilla chinensis* (Bunge) Regel purchased from Anhui Xiehecheng Chinese Herb Limited (23082303); *F. chinensis* Roxb purchased from Anhui Xintai Pharmaceutical Co. Ltd. (221101); *P. chinense* C. K. Schneid and *C. chinensis* Franch purchased from Guizhou tongde pharmaceutical co., LTD. (20230901-01; 20230903-03). These botanical drugs were identified by Professor Zhu Yufeng. The raw botanical drugs *P. chinensis* (Bunge) Regel, *P. chinense* C. K. Schneid, *C. chinensis* Franch and *F. chinensis* Roxb were weighed in a ratio of 5:2:4:4 and decocted twice with ten volumes of water for 30 min each time, and the residue was filtered off. The aqueous solution was passed through 5 layers of medical gauze concentrated to medicinal solution (1 g/mL) and stored at 4°C. DSS is a product of MP Biomedicals (molecular weight: 36,000-50,000, MP Biomedicals, Canada). Salicylazosulfapyridine (SASP) was purchased from Shanghai Xinyitianping Pharmaceutical Co. A LEGENDplex™ Multianalyte Flow Assay Kit was obtained from BioLegend (San Diego, CA, United States). A mouse IL-22 kit was produced by Nanjing Jin Yi Bai Biological Technology Co. Ltd. Carbidopa was purchased from Selleck (S5448), and fetal bovine serum was purchased from New England Limited. Medium was purchased from Sigma-Aldrich (United States). TNF-α was purchased from Novo-protein, L-tryptophan (CAS: 73-22-3), tryptamine (CAS: 61-54-1), 5-hydroxytryptamine (CAS:50-67-9), indole (CAS: 120-72-9) and 3-indole acetic acid (CAS: 87-51-4) were purchased from Sigma (St. Louis, MO, United States), L-kynurenine (CAS: 2922-83-0), indole-3-aldehyde (CAS: 487-89-8) and tryptophol (CAS: 526-55-6) were purchased from Aladdin, methanol and formic acid (LC-MS grade) were obtained from Sigma (St. Louis, MO, United States).

### 2.2 Animals and ethics statement

Seven to eight-week-old female C57BL/6 mice weighing 18–22 g were purchased from the Vital River (Beijing, China) under approval number SCXK (Jing) 2021-0006. Animals were housed under standard conditions (temperature of 23°C ± 2°C, humidity of 50%–70% and a 12-h light/dark cycle) and were fed *ad libitum* with standard chewable pellets and water. Mice were acclimatized for 1 week before the experiment. The handling of the mice and all the experimental procedures were approved by the Experimental Animal Ethics Committee of Jiangsu Provincial Hospital of Traditional Chinese Medicine (2022DW-16-02) and complied with the Guide for the Care and Use of Laboratory Animals.

### 2.3 Induction of UC and experimental design

Acute UC was induced by oral administration of 3.5% (w/vol) DSS in the drinking water for 7 days. The animals were randomized into several groups of 8 mice each. Distilled water was provided to the control mice while 3.5% DSS was given to all the other experimental groups. BTW (6.825 g/kg), SASP (200 mg/kg) or carbidopa (20 mg/kg) were orally administered once a day respectively. Kynurenine (50 mg/kg) was administered intraperitoneally once a day. Clinically, BTW consists of *P. chinensis* (Bunge) Regel (15 g), *P. chinense* C. K. Schneid (12 g), *C. chinensis* Franch (6 g) and *F. chinensis* Roxb (12 g). The adult body weight is 60 kg and the dose conversion factor between human and mouse is 9.1, which translates to a dose of 6.825 g/kg for mice. Mice in the control and model groups received the same volume of vehicle from Day 1 to Day 7.

At the end of the study, whole-blood samples were obtained from the retroorbital venous plexus with a heparinized glass capillary under ether anesthesia. After spinal dislocation, the entire colon was isolated and the length of the colon was measured. The blood samples were centrifuged at 3,000 rpm for 10 min at 4°C to collect the serum for metabolite analysis. The mice colons were preserved, one part was fixed for 24 h in 4% paraformaldehyde, and the other part was stored at −80°C for further experiments.

### 2.4 Assessment of DAI score

The DAI was calculated according to the methods of a previous study (Sann et al., 2013).

### 2.5 Histological and immunohistochemical analysis

Colon sections were rinsed with ice-cold PBS, the excess PBS was removed, and the tissues were immediately fixed in 4% paraformaldehyde. Sections were stained with H&E, AB-PAS, Ki67, ZO-1 and Occludin respectively.

### 2.6 Inflammatory factors detect

All the inflammatory factors in this study were detected in mice serum using a LEGENDplex™ Multi-Analyte Flow Assay Kit.

### 2.7 Protein analysis and quality control

Sample preparation: Mice colon tissue samples were grounded into cellular powder using liquid nitrogen and then transferred to centrifuge tubes. After that, four volumes of lysis buffer (8 M urea, 1% protease inhibitor cocktail) were added to the cell powder, followed by sonication three times on ice using a high intensity ultrasonic processor. The remaining debris was removed by centrifugation at 12,000 g at 4°C for 10 min. Finally, the supernatant was collected and the protein concentration was determined with BCA kit according to the manufacturer’s instructions.

Trypsin Digestion: For digestion, the protein solution was reduced with 5 mM dithiothreitol for 30 min at 56°C and alkylated with 11 mM iodoacetamide for 15 min at room temperature in the dark. The protein sample was then diluted by adding 100 mM TEAB to a urea concentration less than 2 M. Finally, trypsin was added at a 1:50 trypsin-to-protein mass ratio for the first digestion overnight and a 1:100 trypsin-to-protein mass ratio for a second 4 h-digestion. Finally, the peptides were desalted on a C18 SPE column.

LC‒MS/MS analysis: The peptides were separated using a Nano Elute ultra-high performance liquid chromatography (UHPLC) system after being solubilized with liquid chromatography liquid-phase A. liquid-phase A was an aqueous solution containing 0.1% formic acid and 2% acetonitrile; liquid-phase B was a solution containing 0.1% formic acid and 100% acetonitrile. The liquid-phase gradient settings were as follows: 0–70 min, 6%–24% B; 70–82 min, 24%–35% B; 82–86 min, 35%–80% B; and 86–90 min, 80% B. The flow rate was maintained at 450 nL/min. The peptides were separated by an ultrahigh performance liquid chromatography (UPLC) system, injected into a capillary ion source for ionization and subsequently analyzed via mass spectrometry (MS) with a TOF Pro 2. The ion source voltage was set at 1.6 kV, and the peptide parent ion and its secondary fragments were detected and analyzed by high-resolution TOF. The scanning range of the secondary mass spectra was set to 100–1700.The data acquisition mode used was parallel accumulated serial fragmentation (PASEF) mode. One primary mass spectrum was acquired followed by 10 secondary spectra in PASEF mode with parent ion charges in the range of 0–5, and the dynamic exclusion time of the tandem mass spectrometry scans was set to 30 s to avoid repeated scans of the parent ions.

### 2.8 Targeted metabolite analysis

#### 2.8.1 Sample processing

Fifty microliters of thawed mouse serum samples were aspirated into clean EP tubes, 150 µL of pure methanol containing d5-phenylalanine isotope internal standard (5 μg/L) was added for extraction, the mixture was vortexed for 5 min to ensure good mixing, the solution was centrifuged at 18,000 rpm for 10 min to precipitate the protein, and 150 µL of the supernatant was pipetted into new EP tubes and blown dry with nitrogen. Then, 100 µL of 50% acetonitrile solution was added to the dried sample, which was vortexed for 5 min to mix well and centrifuged at 12,000 rpm for 10 min, after which 60 μL of the supernatant was aspirated into the injection vial. The colon tissue was cut, 20 mg of colon content was weighed in a clean EP tube, 400 µL of pure methanol containing the d5-phenylalanine isotope internal standard (5 μg/mL) was added, the mixture was vortexed for 5 min, and the mixture was centrifuged for 10 min at 12,000 rpm to precipitate the proteins. Afterward, 300 µL of the supernatant was aspirated to a new EP tube, after which the mixture was blown dry with nitrogen gas. The following steps were the same as those for the serum.

#### 2.8.2 Instrument conditions

Liquid chromatographic procedure: The column used was a Welch Ultimate XB-C18 column (2.1 mm × 100 mm, 5 μm). The liquid-phase consisted of liquid A (0.01% water) and liquid B (acetonitrile). The samples were placed in an autosampler at 8°C, the column temperature was 30°C, the flow rate was 500 μL/min and the injection volume was 1 μL. The relevant liquid-phase gradients were as follows: from 0 to 3 min, Liquid B was the 10% line; from 3.1 to 9 min, Liquid B was 100%; and from 9.1 to 15 min, Liquid B was 10%. The sample cohort was set up with one QC sample per interval of a certain number of experimental samples for the detection and evaluation of the stability and repeatability of the system.

### 2.9 *In vitro* research

#### 2.9.1 Cell culture and treatment

Caco-2 cells (American Type Culture Collection, Rockville, MD) were cultured in MEM supplemented with 20% fetal bovine serum. Log phase growth cells were taken during cell administration and inoculated in six-well plates (1.5 × 10^5^ cells/well), and the fresh culture medium was changed every other day. The cells were randomly grouped, and TNF-α (50 ng/mL) was given to the experimental group for modeling, and the corresponding volume of PBS was added to the control group. Lyophilized powder of BTW was prepared into a 40 mg/mL master solution with DMSO, and low, medium and high concentrations of BTW were prepared as 10, 20, and 40 μg/mL, which were dissolved in the MEM medium at the same time as TNF-α to intervene for 24 h. IET (0.0125, 0.025, and 0.05 mM), I3A (0.0125, 0.025, and 0.05 mM) and carbidopa (30 μM) were administered to the cells in the same manner as BTW. Cells were rinsed, scraped, and collected for protein blot analysis or RNA extraction for qPCR using RNA extraction kit.

#### 2.9.2 CCK-8 assay

CCK-8 assay was performed using a CCK-8 Assay Kit (Yesen Bio, China) according to the manufacturer’s instructions. Optical density (OD) was measured at 450 nm using a BioTek 800 TS enzyme labeler (Agilent, United States).

#### 2.9.3 RNA extraction and qPCR

The extraction of total RNA from Caco-2 cells and the detection of each gene were consistent with previous studies (Wei et al., 2023). The primer sequences are shown in Table 1.

**TABLE 1:** qPCR primer sequences.

Gene	Forward primer sequence (5′–3′)	Reverse primer sequence (5′–3′)
GAPDH	CTG​TGC​CCA​TCT​ACG​AGG​GCT​AT	TTT​GAT​GTC​ACG​CAC​GAT​TTC​C
IL-6	ACT​CAC​CTC​TTC​AGA​ACG​AAT​TG	CCA​TCT​TTG​GAA​GGT​TCA​GGT​TG
IFN-γ	TCG​GTA​ACT​GAC​TTG​AAT​GTC​CA	TCG​CTT​CCC​TGT​TTT​AGC​TGC

### 2.10 Western blot analysis

Total proteins from colon tissues and cells were extracted and analyzed by the following antibodies: GAPDH (proteintech, #HRP-60004), ZO-1 (abcam, #ab216880), Occludin (abcam, #ab216327), MUC2 (abcam, #ab272692), Bcl-2 (proteintech, #68103-1-Ig), Bax (proteintech, #60267-1-Ig), AHR (proteintech, #67785-1-Ig), CYP1A1 (ABclonal, #A2159), Tubulin (proteintech, #11224-1-AP) and DDC (ABclonal, #A3828). The experiments were following as previous studies (Wei et al., 2023).

### 2.11 Statistics analysis

The experimental data are expressed as mean ± SD. Two-way ANOVA was used to assess the significance of differences in body weights and DAI. One-way ANOVA was used to evaluate the significance of differences between multiple groups for one variable. *p* < 0.05 was considered to indicate a statistically significant difference. The data were analyzed and plotted using GraphPad Prism 9.5 software (GraphPad, San Diego, CA, United States).

## 3 Results

### 3.1 BTW ameliorates colitis development in DSS-induced UC mice

**FIGURE 1:**
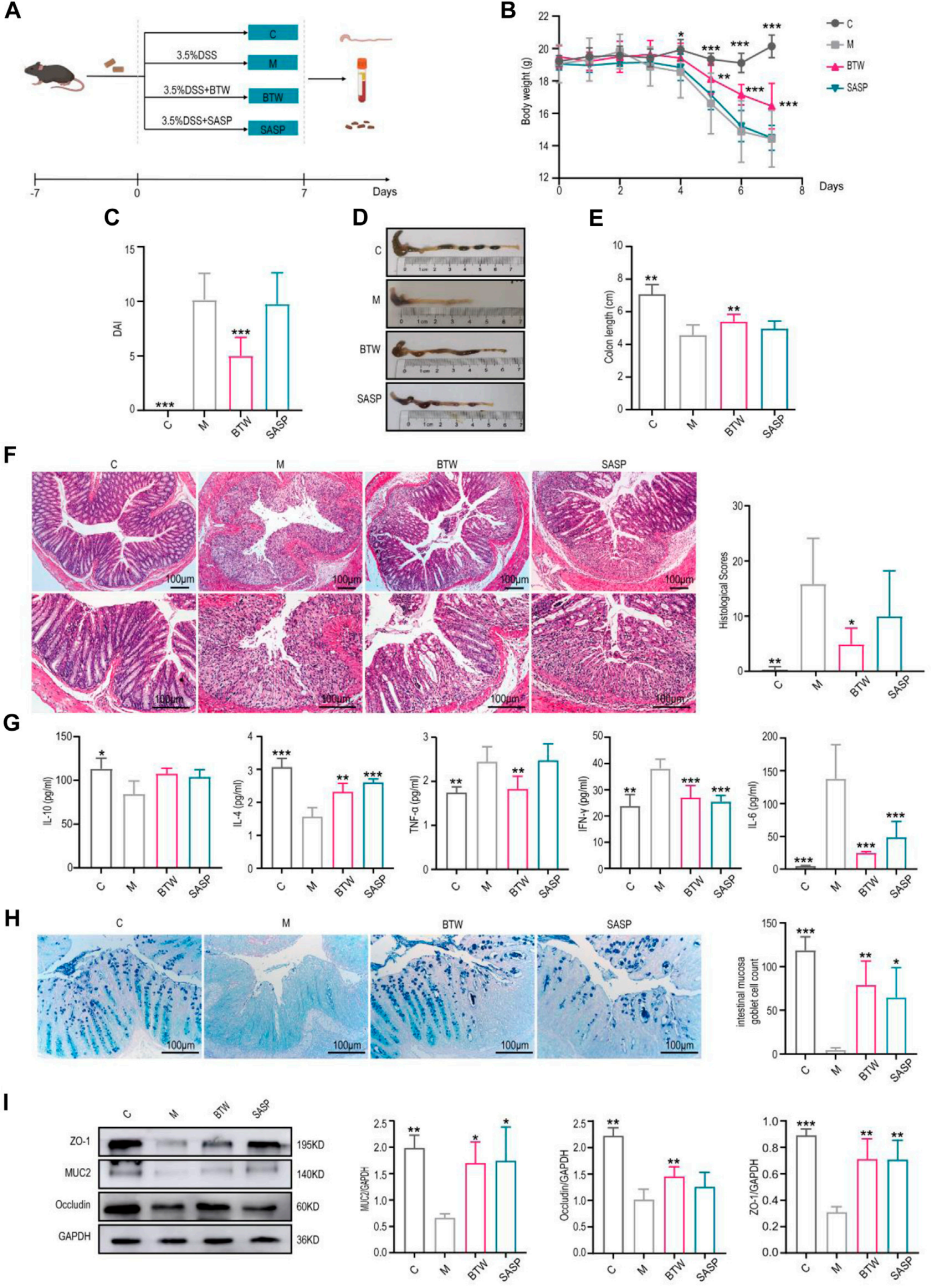
BTW ameliorates colitis development in DSS-induced UC mice. **(A)** Flow chart of experimental animals (*n* = 8). **(B)** Body weight changes of mice throughout the entire trial (*n* = 8). **(C)** DAI scores of the mice in each group on Day 7 (*n* = 8). **(D)** Representative images of colons. **(E)** Statistical analysis of colon length in each group (*n* = 8). **(F)** H&E staining of the colon (scale bar: 100 μm, *n* = 6). **(G)** Flow cytometry analysis of the serum expression levels of IL-10, IL-4, TNF-α, IFN-γ and IL-6 (*n* = 3–6). **(H)** Representative AB-PAS staining and intestinal mucosa goblet cell count (*n* = 6). **(I)** Western blot analysis of intestinal barrier protein expression in colon tissues (*n* = 3). **(C)** Control, M: DSS-induced Model. ^*^ *p* < 0.05, ^**^ *p* < 0.01, ^***^ *p* < 0.001 vs. Group M; data are expressed as the mean ±.

DSS-induced colitis is the most widely used experimental murine model of UC (Katsandegwaza et al., 2022). In our study, mice were treated with 3.5% DSS for 7 days to establish a UC model (Figure 1A). In model group, the body weight of mice decreased gradually from Day 4 until Day 7. Compared with the model group, the body weight in the BTW group improved after Day 4 (Figure 1B). The DAI score (including rate of weight loss, fecal consistency and blood in the stool) was significantly increased in model group, while BTW treatment significantly decreased the DAI score (Figure 1C). Colon shortening is positively associated with colonic inflammation and edema in DSS-induced colitis. DSS treatment significantly shortened the colon length, whereas BTW significantly extended the colon length (Figures 1D,E). SASP also increased colon length with less improvement than BTW. In model group, mucosal layer of mice was seriously damaged, and significant inflammatory infiltration was observed by H&E staining. In comparison, the BTW treatment significantly improved the mucosal layer and alleviated inflammatory infiltration (Figure 1F). In addition, the level of major pro-inflammatory cytokines interleukin-6 (IL-6), interferon gamma (IFN-γ), and tumor necrosis factor-alpha (TNF-α) were increased, while the levels of the anti-inflammatory cytokine interleukin-10 (IL-10) and interleukin-4 (IL-4) were decreased in model group. BTW and SASP treatment markedly suppressed the elevation of IL-6, IFN-γ and TNF-α, while they elevating the level of IL-10 and IL-4 (Figure 1G).

DSS-induced colitis was accompanied by a decrease of goblet cells and mucin secretion, and the number of goblet cells and mucin secretion were greater in the treatment groups than in the model group (Figure 1H). We found that BTW significantly upregulated the expression of MUC2 in colon tissues. The BTW treatment also effectively improved the intestinal barrier damage caused by DSS, which was indicated by the significantly increased expression of Occludin and ZO-1 (Figure 1I). The expression levels of the proapoptotic protein Bax was significantly greater in the model group than in the control group, and was effectively suppressed by the BTW or SASP treatments. In contrast, the expression of antiapoptotic protein Bcl-2 was decreased in the model group, and the BTW and SASP treatments counteracted this decrease of Bcl-2 respectively (Supplementary Figure S1A). Moreover, the expression of proliferation maker Ki67 was significantly lower in the model group than in the control group, while BTW treatment strongly made these elevations (Supplementary Figure S1B). It should be noted that SASP group can also alleviate these symptoms, but the therapeutic effect is not as good as BTW.

### 3.2 BTW regulates disorders of Trp metabolism in UC mice

To gain further insight into the mechanism of BTW-treated UC, we performed proteomic analysis of colon tissues from control, model and BTW groups of mice. Pearson’s correlation coefficient (PCC) and Principal component analysis (PCA), were used to assess the reproducibility (Supplementary Figure S2). The summary data of all differentially expressed proteins in this project are detailed in Table 2. Compared with the control group, the model group had 451 downregulated and 436 upregulated proteins. Compared with the BTW group, the model group had 225 downregulated and 183 upregulated proteins.

**TABLE 2:** Statistical information on proteomics differential proteins.

Comparison group	Upward (>1.5)	Downward (<1/1.5)
M/C	436	451
M/BTW	183	225

**FIGURE 2:**
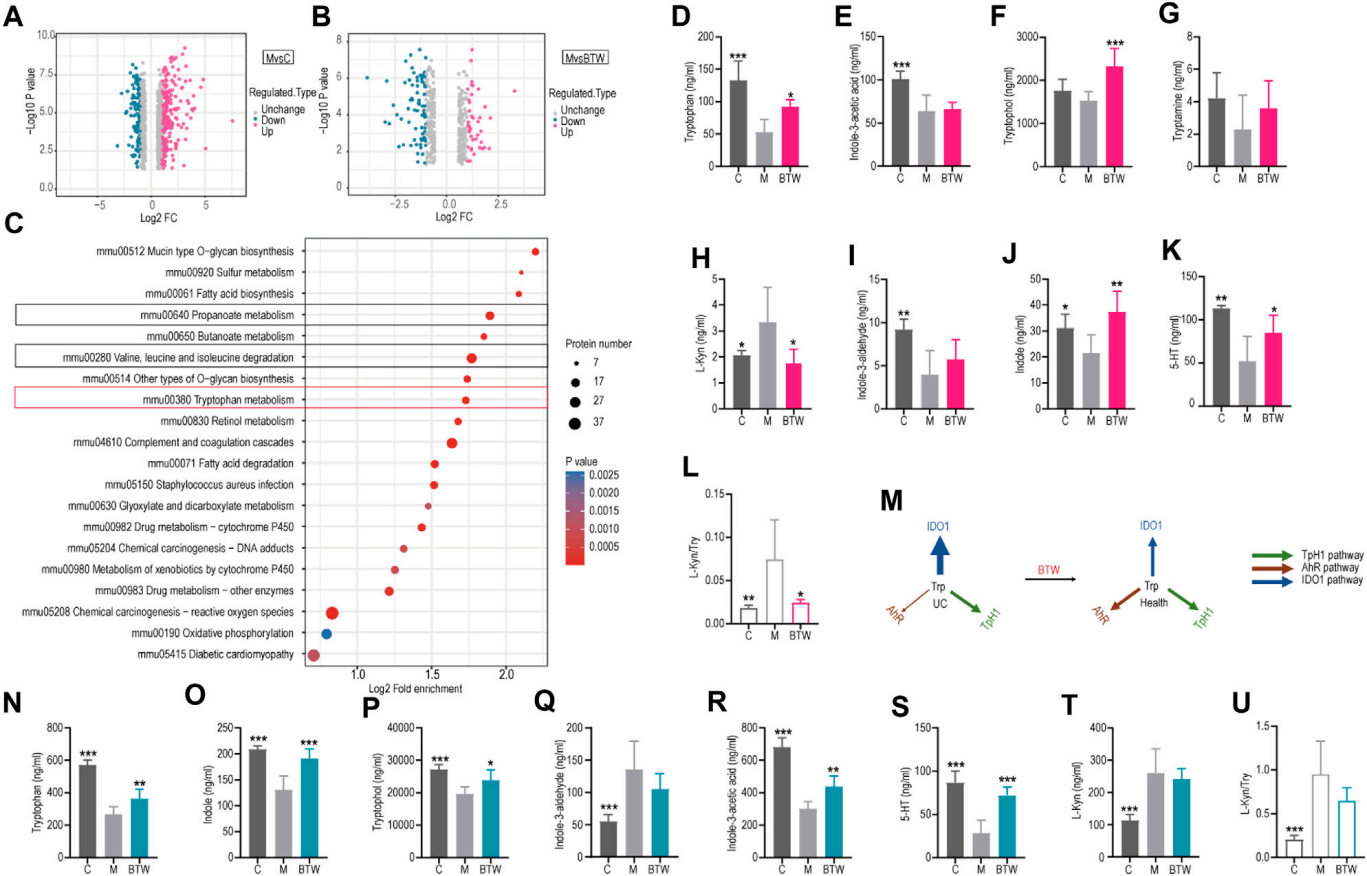
BTW regulates disorders of tryptophan metabolism in UC mice. **(A)** Volcano plot of differentially expressed proteins in the M/C; **(B)** M/BTW Volcano plot of differentially expressed proteins **(C)** Analysis of Control/Model KEGG enrichment via proteomics in mouse colon tissue. **(D)** Tryptophan (Trp), **(E)** indole -3-acetic acid, **(F)** tryptophol, **(G)** tryptamine, **(H)** kynurenine (Kyn), **(I)** indole-3-aldehyde, **(J)** indole, **(K)** 5-HT and **(L)** Kyn/Try content analysis of normal and UC mouse serum (*n* = 5–6). **(M)** Distribution of tryptophan metabolic pathways pathway metabolites in UC mice. **(N)**Tryptophan, **(O)** indole, **(P)** tryptophol, **(Q)** indole-3-aldehydeIndole, **(R)** indole -3-acetic acid, **(S)** 5-HT, **(T)** Kyn, **(U)** Kyn/Try content analysis in colon tissues (*n* = 6). ^*^ *p* < 0.05, ^**^ *p* < 0.01, ^***^ *p* < 0.001 vs. group M; ^#^ *p* < 0.05, ^##^ *p* < 0.01, ^###^ *p* < 0.001 vs. BTW Group; data are expressed as the mean ± SD.

As shown in Figures 2A,B, a volcano plot was used to display the information of differential proteins. The horizontal axis of the plot was the value of the protein differential fold change (FC) value after Log2 logarithmic transformation, and the vertical axis was the value of the significance of difference test *p*-value after -Log10 logarithmic transformation. Red points in the graph indicated different proteins with significantly upregulated expression, and blue points indicated different proteins with significantly downregulated expression. KEGG signaling pathway analysis was performed on the differentially expressed proteins, and the results indicated that the signaling pathways involved were mainly related to the degradation of valine, leucine, and isoleucine; short-chain lipid metabolism and Trp metabolism (Figure 2C). Trp and its metabolites play vital roles in the regulation of intestinal inflammation by acting directly or indirectly on the pro/anti-inflammatory cytokines, functions of various immune cells, as well as the intestinal microbial composition and homeostasis (Gao et al., 2018; Li et al., 2021). Given the close correlation between Trp metabolism and UC, we further investigated the significance of BTW in Trp metabolism.

Targeted metabolomics analyses were performed to analyze the serum and colon from the different groups of mice with colitis, after which we attempted to characterize the differential metabolites that were implicated in the development of DSS-induced colitis. Eight Try metabolites were detected in the serum, namely, Try (Figure 2D), I3A (Figure 2E), IET (Figure 2F), tryptamine (Figure 2G), kynurenine (Figure 2H), indole-3-aldehyde (Figure 2I), indole (Figure 2J) and 5-hydroxytryptamine (5-HT) (Figure 2K). Compared to those in the control group, the model group had significantly greater levels of kynurenine, but lower levels of Try, resulting in an increase in the ratio of kynurenine to Try (Figure 2L). Moreover, the levels of all the other metabolites decreased. However, BTW reversed these alterations, and IET, indole and 5-HT showed more significant improvements after BTW treatment. Targeted metabolomics analysis of the mouse colon almost showed the similar results with serum (Figures 2N–U). In UC clinical patients and colitis mice, the metabolic pathways of Trp metabolism are dysregulated, with an increase in the kynurenine metabolic pathway and a decrease in the indole metabolic pathway (Borren et al., 2021; Cheng et al., 2022). The various metabolic pathways involving tryptophan are also relatively dynamic (Agus et al., 2018). As revealed by targeted metabolomics, BTW administration could modulate Trp metabolism and promote the conversion of kynurenine metabolites to indole metabolites in colitis mice (Figure 2M).

### 3.3 Kynurenine accumulation counteracts the improvement in intestinal barrier function in colitis mice induced by BTW

**FIGURE 3:**
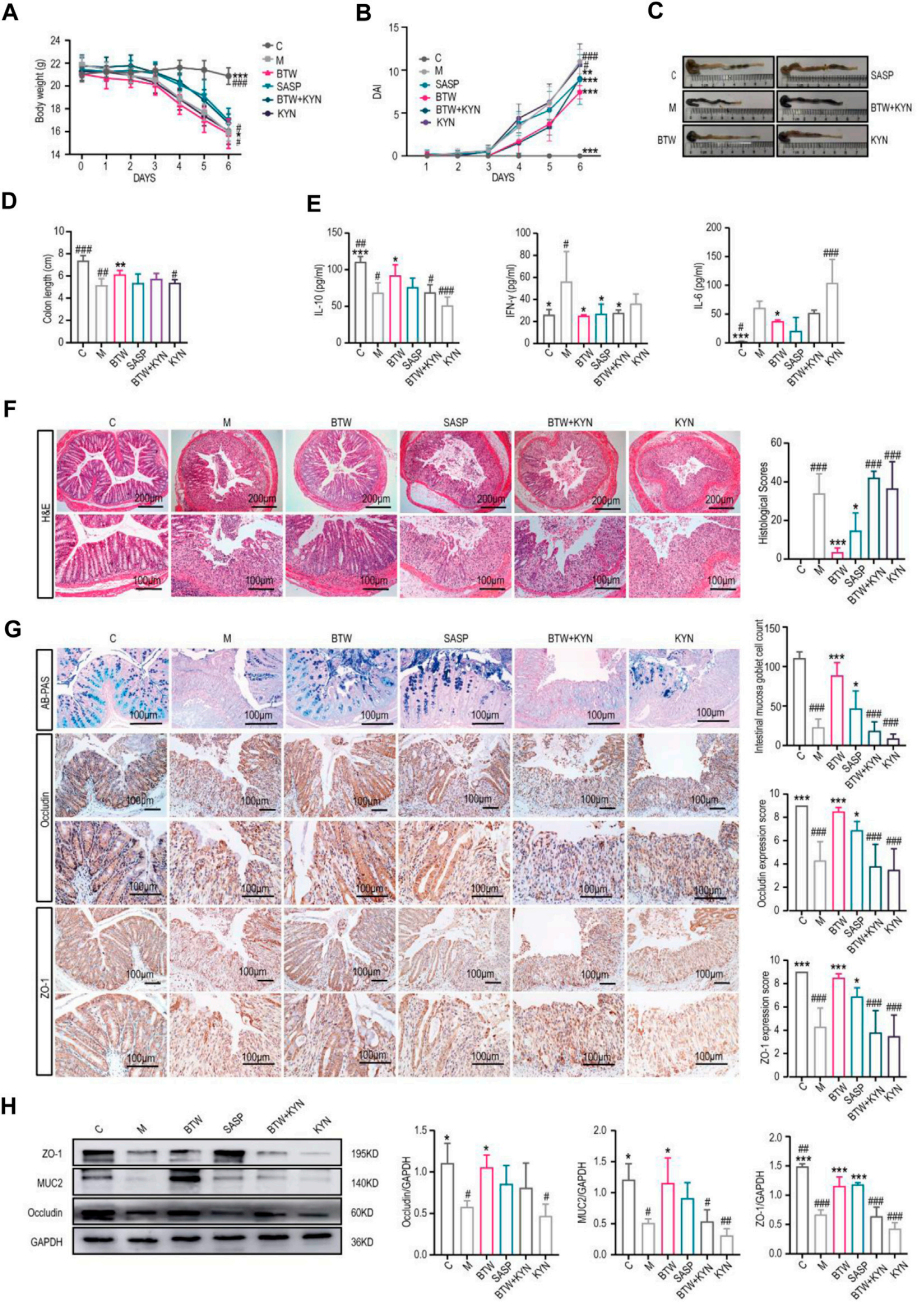
Kynurenine accumulation counteracts the improvement of intestinal barrier function in UC mice induced by BTW. **(A)** Body weight changes of mice throughout the entire trial (*n* = 8). **(B)** DAI score from Day 1-6 in each group. **(C)** Macroscopic appearance of the colon. **(D)** Quantitative measurement of colon length (*n* = 8). **(E)** Flow cytometry was used to determine the expression levels of IL-10, IFN-γ and IL-6 in mouse serum (*n* = 4). **(F)** Representative H&E stained sections (scale bar: 200 μm/100 μm, *n* = 3). **(G)** Representative AB-PAS staining (scale bar: 100 μm, *n* = 5); immunohistochemistry images and expression scores of Occludin and ZO-1and the expression of intestinal barrier proteins in the colonic tissues (*n* = 5). **(H)** Western Blot analysis of intestinal barrier protein expression in colon tissues (*n* = 3). BTW + KYN: BTW and kynurenine coadministration group; KYN: kynurenine group. ^*^ *p* < 0.05, ^**^ *p* < 0.01, ^***^ *p* < 0.001 vs. Group M; ^#^ *p* < 0.05, ^##^ *p* < 0.01, ^###^ *p* < 0.001 vs. BTW Group; the data are expressed as the mean ± SD.

To confirm whether the protective effect of BTW against UC is dependent on a reduction in kynurenine levels, we evaluated the effects of kynurenine on DSS-induced acute colitis in mice *in vivo*. The results indicated that the model group and kynurenine group had lower body weights, greater DAI scores, and shorter colons compared with the BTW group, and these symptoms were alleviated by BTW and SASP. However, the improvement effect of BTW was abolished by kynurenine (Figures 3A–D). The results of the multifactorial kit showed that the levels of proinflammatory cytokines (IL-6 and IFN-γ) were upregulated and the level of an anti-inflammatory cytokine (IL-10) was downregulated in model group. BTW upregulated IL-10, downregulated IL-6 and IFN-γ, but kynurenine treatment abrogated the effect of BTW (Figure 3E).

Histological analysis of the mice revealed that after the administration of kynurenine, the weight of the mice decreased significantly, while BTW and SASP treatment significantly alleviated these pathological changes (Figure 3F). Kynurenine reversed the beneficial effect of BTW in DSS-treated mice, as indicated by increased tissue damage, increased inflammatory infiltration and decreased expression of TJ proteins (ZO-1, Occludin) (Figures 3F–H). The number of Ki67-positive cells in each crypt was reduced, while Bax expression was upregulated and Bcl-2 expression was downregulated in the group with kynurenine involvement compared to the BTW group (Supplementary Figure S3). These data confirmed the role of kynurenine in reducing the protective effect of BTW against UC.

### 3.4 BTW activates the AHR/IL-22 signaling pathway through upregulating DDC

**FIGURE 4:**
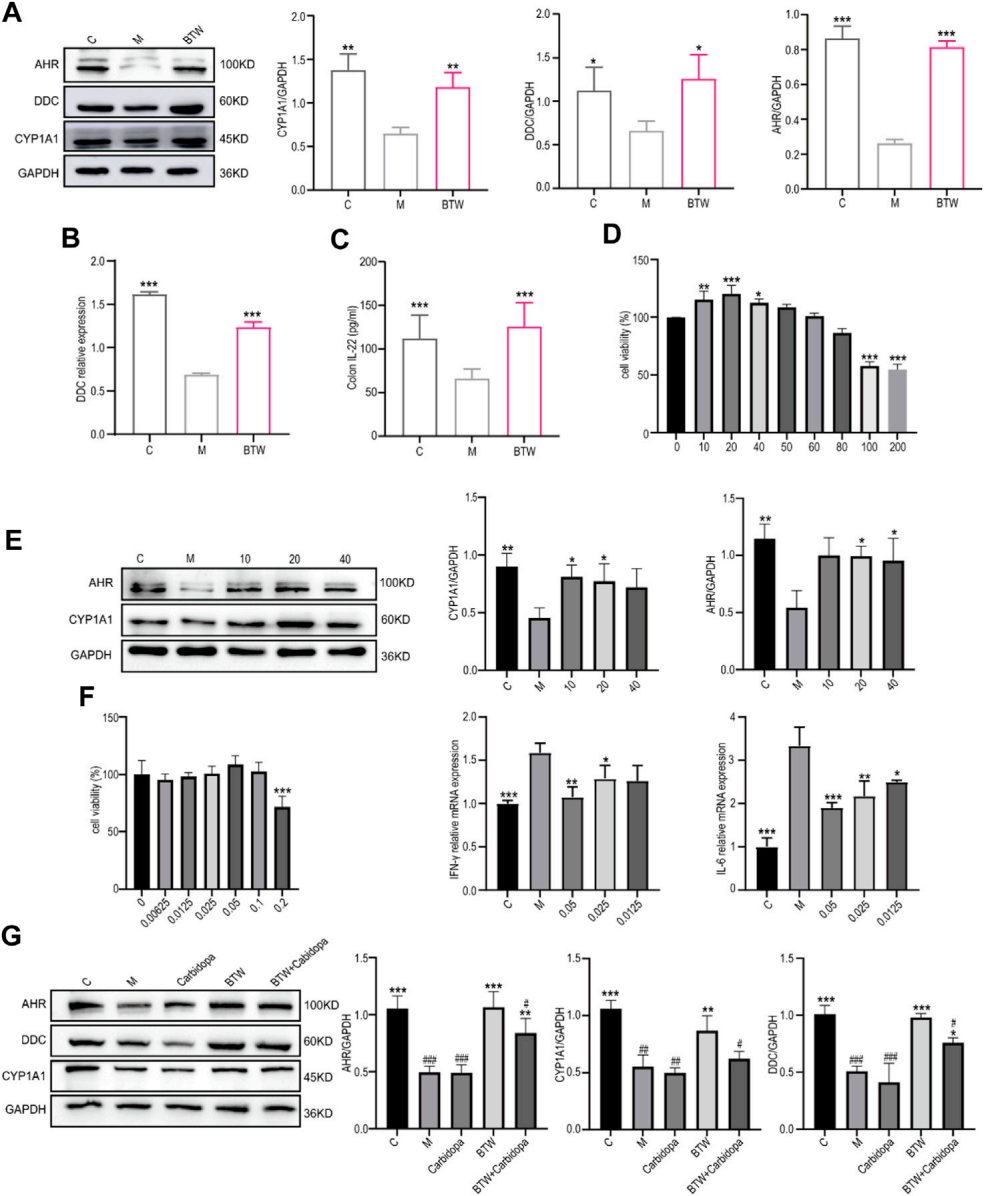
BTW activates the AHR/IL-22 signaling pathway through upregulating DDC. **(A)** Western blot analyses of the AHR pathway in the mouse colon (*n* = 3). **(B)** Quantification of relative expression of DDC in the proteomic analysis (*n* = 3). **(C)** IL-22 levels in the mouse colon were detected via ELISA (*n* = 4). **(D)** The CCK8 assay was used to detect the viability of Caco-2 cells by BTW. **(E)** Western blot analysis of AHR pathway proteins in Caco-2 cells after BTW administration (*n* = 3). **(F)** CCK8 was used to detect the viability of Caco-2 cells by IET and qPCR analysis of the relative expression levels of IL-6 and IFN-γ. (*n* = 3). **(G)** Western blot analyses of AHR pathway in mice colon after carbidopa administration (*n* = 3). ^*^ *p* < 0.05, ^**^ *p* < 0.01, ^***^ *p* < 0.001 vs. Group M; ^#^ *p* < 0.05, ^##^ *p* < 0.01, ^###^ *p* < 0.001 vs. BTW Group; the data are expressed as the mean ± SD.

AHR serves as a ligand-activated transcription factor playing role in shaping the innate and adaptive immune responses. Indole metabolites were described as a gut protector due to the activation effect on the AHR receptor (Scott et al., 2020). To analyze the regulatory effect of BTW on indole metabolism, we detected the levels of AHR and CYP1A1 (indicator of AHR activation) in mouse colon tissue after BTW treatment. AHR and CYP1A1 were significantly downregulated in DSS-induced colitis mice, whereas they were significantly upregulated by BTW treatment (Figure 4A). DDC is an important metabolic enzyme in Trp metabolism with tryptamine, IET and I3A were the downstream metabolites. Both the results of western blot (Figure 4A) and proteomics (Figure 4B) indicated that the relative expression levels of DDC were decreased in the colon of UC mice, but were significantly increased after BTW treatment. AHR activation-induced IL-22 production is important for maintaining epithelial cell integrity (Meynier et al., 2022; Zhao et al., 2023). The ELISA results showed that IL-22 was downregulated in colon tissue from the model group, while BTW reversed the DSS-induced reduction in IL-22 (Figure 4C).

To further determine the effect of BTW on the AHR/IL22 pathway, the human colon cancer cell line Caco-2 (Liu et al., 2021; Yang et al., 2023a) was used for *in vitro* analysis. The cytotoxicity of BTW at the indicated concentrations was excluded (Figure 4D), and TNF-α was chosen to induce inflammation (Huang et al., 2020). Western blot analyses demonstrated that TNF-α obviously decreased the levels of AHR and CYP1A1 in comparison with the control group, while BTW treatment significantly increased the protein levels of AHR and CYP1A (Figure 4E). The metabolites IET and I3A, which are the downstream of Trp metabolized by DDC, was found to reduce the mRNA levels of IL-6 and INF-γ in an inflammation model induced by TNF-α (Figure 4F, Supplementary Figure S4A). But tryptamine, which is metabolized by DDC as the first metabolite of tryptophan, had no significant effect (Supplementary Figure S4B). To verify the role of DDC in the regulation of Trp metabolism by BTW, the DDC inhibitor carbidopa was applied. As shown in Figure 4G, carbidopa partially abolished the regulatory effect of BTW on the AHR/IL22 pathway. These data suggested that BTW may exert its anti-colitis effect by regulating tryptophan catabolic metabolites through the promotion of DDC expression.

### 3.5 BTW improves intestinal barrier function through DDC regulation

**FIGURE 5:**
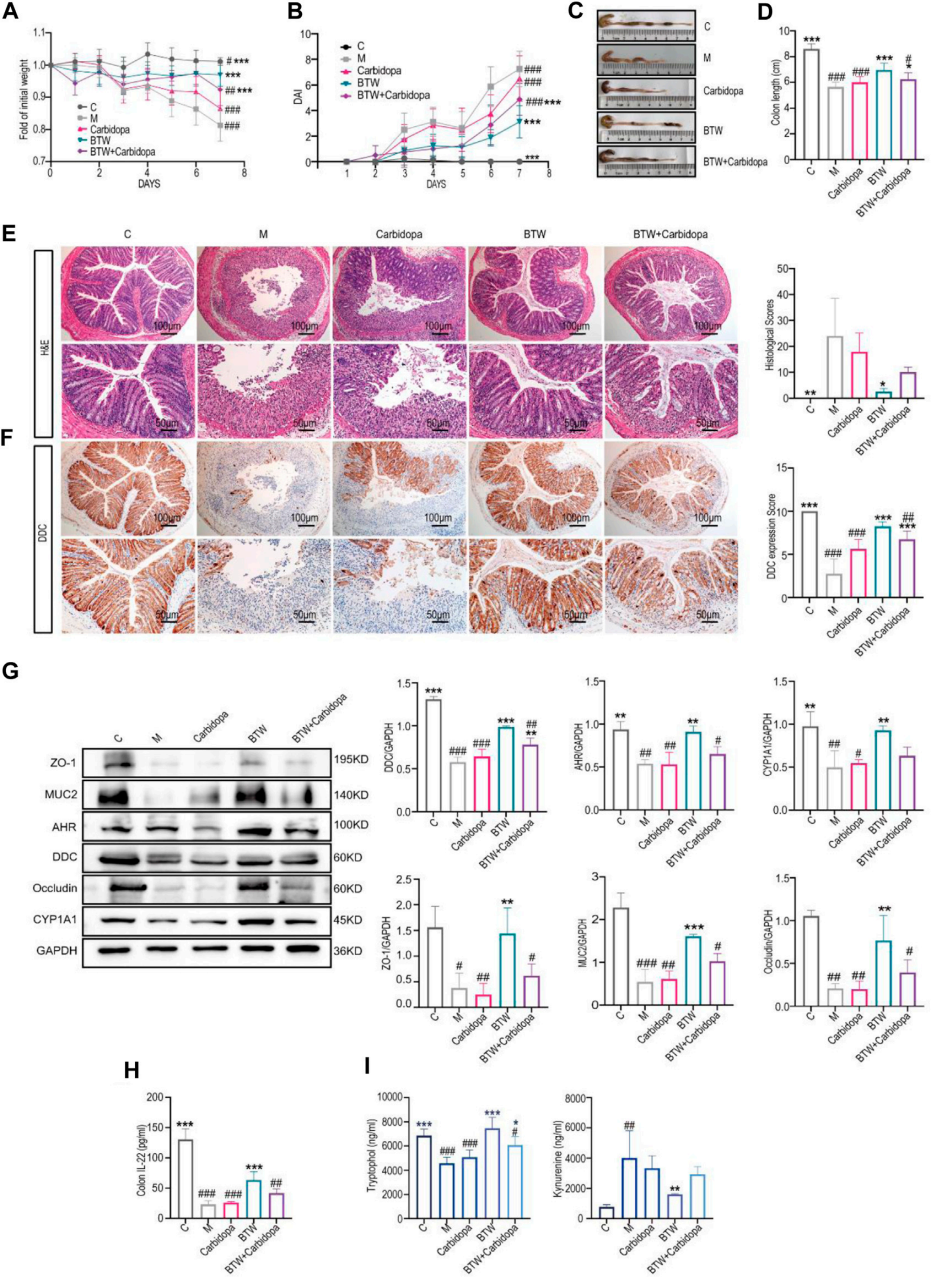
BTW improves intestinal barrier function through DDC regulation. **(A)** Body weight changes of mice throughout the entire trial (*n* = 8). **(B)** DAI scores of mice in each group from Day 1–7 (*n* = 8). **(C)** Macroscopic appearance of the colon. **(D)** Quantitative measurements of the length of the colon (*n* = 8). **(E)** Representative H&E-stained sections (scale bar: 200 μm/100 μm, *n* = 3). **(F)** Immunohistochemical assays showed DDC levels in colon tissues (scale bar: 200 μm, *n* = 3). **(G)** Western blot analyses of the AHR pathway, TJ proteins and MUC2 proteins in mice colon (*n* = 3). **(H)** ELISA analyses IL-22 content of the mice colon (*n* = 8). **(I)** IET and kynurenine content analysis in different groups (*n* = 4). ^*^ *p* < 0.05, ^**^ *p* < 0.01, ^***^ *p* < 0.001 vs. Group M; ^#^ *p* < 0.05, ^##^ *p* < 0.01, ^###^ *p* < 0.001 vs. BTW Group; data are expressed as mean ± SD.

To further confirm the role of DDC in the regulation of UC by BTW, we administered carbidopa to DSS-induced colitis mice. The results indicated that carbidopa treatment abolished the therapeutic effect of BTW, as indicated by a reduced body weight (Figure 5A), an upregulated colonic DAI (Figure 5B), a reduced colon length (Figures 5C,D) and increased inflammatory cell infiltration and epithelial cell shedding in the colon tissue (Figure 5E). The expression of IL-6 and IFN-γ were downregulated in the model group and carbidopa group. The therapy effect of BTW + carbidopa group was much less significant than that in the BTW group (Supplementary Figure S5). Immunohistochemical staining showed that carbidopa inhibited DDC expression and BTW partially promoted DDC expression (Figure 5F).

The expression of colonic ZO-1, Occludin and MUC2 was significantly lower in the model group and carbidopa group, whereas it was significantly greater in the BTW-treated group. However, the positive effects of BTW were significantly suppressed after carbidopa administration. Immunohistochemical analyses of ZO-1 and Occludin provided consistent results (Figures 5G, Supplementary Figure S6). The expression of colonic CYP1A1 and AHR were downregulated in the carbidopa and model groups, respectively, whereas the effect of BTW on the regression of these two proteins was disrupted by the addition of carbidopa (Figure 5G). IL-22 level was significantly reduced in DSS-induced colitis mice, and BTW treatment significantly increased the concentration of IL-22 in colitis mice (Figure 5H). However, the concentration of IL-22 in the BTW + Carbidopa group was significantly lower than that in the BTW group. As shown in Figure 5I, DDC inhibition increased the level of kynurenine but decreased IET, which abrogated the effect of BTW. All the results indicated that BTW could ameliorate Trp metabolism in UC by upregulating DDC to activate AHR/IL-22 pathway.

## 4 Discussion

In recent years, the incidence of UC has increased globally, especially in developing countries (Da Luz Moreira et al., 2022), therefore, there is an urgent need to develop effective drugs for the treatment of such diseases. BTW is widely used as a traditional prescription drug for the treatment of UC in China. However, the underlying therapeutic mechanism has not been fully elucidated. Previous research revealed that BTW alleviates experimental colitis by suppressing the IL-6/STAT3 signaling pathway, modulating the Th17/Treg cell balance, and improving microflora structure and bile acid metabolisms (Monteleone et al., 2011; Ma et al., 2018; Wei et al., 2023). In the present study, we attempted to clarify the underlying mechanisms, the alleviating effect of BTW on UC was shown to be related to tryptophan metabolism and it was confirmed how BTW regulates tryptophan metabolism.

In this study, as a basic study, we followed the 4R rules of using as few animals as possible. we provided evidence that BTW can largely improve colitis symptoms and restore intestinal barrier function. Quantitative proteomic study of UC mice colon tissues was carried out through the organic combination of a series of cutting-edge technologies, such as protein extraction, enzymatic digestion, liquid chromatography-mass spectrometry tandem analysis, and bioinformatic analysis. KEGG pathway of proteomics analysis indicated that tryptophan metabolic signaling pathway was significantly enriched in DSS-induced colitis mice. We analyzed the changes of eight tryptophan metabolites in serum and colon from different colitis mice groups using Targeted Metabolomics analysis. It suggested that BTW could modulate disorders of Trp metabolism through decreasing kynurenine accumulation, and increasing indole metabolites. Also, our data indicated that kynurenine exacerbated intestinal damage and abolished the effects of BTW in UC mice. Finally, we verified that BTW affects tryptophan metabolites through increasing DDC expression, which activates the AHR/IL-22 pathway and improves the intestinal barrier.

A variety of bioactive compounds produced through Trp metabolism can modulate various physiological functions, including inflammation, metabolism, immune responses (Gasaly et al., 2021), and neurological functions (Li et al., 2022). Trp is involved in three metabolic pathways, the kynurenine pathway, indole metabolism pathway and serotonin metabolism pathway. New research supports a close relationship between tryptophan metabolic disorders and UC (Shi et al., 2024). Many indole derivatives in the intestine, such as indole-3-aldehyde, I3A, indole-3-propionic acid, indole-3-acetaldehyde, and indole acrylic acid, are ligands for AHR (Alexeev et al., 2018). In the procedure of Trp metabolism, DDC may catabolize tryptophan to metabolites such as tryptamine, I3A and IET, whereas these metabolites have been reported to act as ligands for the AHR and to stimulate downstream target gene IL-22 epitopes, which improves intestinal barrier function (Zelante et al., 2013; Agus et al., 2018). Therefore, the disorder of DDC may impede the production of indole metabolites further influence the AHR/IL-22 pathway. In this work, we confirmed that BTW was a DDC enhancer to elevate the expression of DDC, which promoted the production of I3A and IET and activated the AHR/IL-22 pathway. The protective effect was abolished by carbidopa, a DDC inhibitor, which was applied for Parkinson’s disease in clinic.

BTW consists of *P. chinensis* (Bunge) Regel, *P. chinense* C. K. Schneid, *C. chinensis* Franch and *F. chinensis*. BTW has a good therapeutic effect on UC mice, and its pharmacological mechanism could be associated with the maintaining of homeostasis and diversity of intestinal flora, increasing the content of short-chain fatty acid (SCFAs), and repairing the colonic mucosal barrier, etc. (Hua et al., 2021; Niu et al., 2023; Pan et al., 2024) In our published results, by comparing the retention time of the standards, ten chemical metabolites were identified from the total ion chromatogram of BTW. Esculetin, Esculin and Anemoside B4 were detected under negative ion mode, while Fraxetin, Palmatine chloride, Limonin, Epiberberine, Phellodendrine chloride, Berberine chloride and Coptisine were identified in positive ion mode. Berberine, as an important metabolite of BTW, has been extensively studied as an anti-autoimmune agent and has been reported to be a promising candidate for the treatment of UC (Li et al., 2016; Yang et al., 2023b). Anemoside B4 could prevent chronic relapsing colitis in mice by modulating the inflammatory response, the colonic transcriptome, and the gut microbiota (Han et al., 2022; Feng et al., 2023; Zhao et al., 2023). It is still worth exploring whether BTW works through a specific metabolite, and whether the effect of formula is more moderate than that of single metabolite.

In addition, UC is associated with dysbiosis of normal flora (Wang et al., 2020; Liu et al., 2022; Niu et al., 2022). Meanwhile, Williams Brianna b, et al. has indicated that two bacteria from the Phylum Firmicutes contain tryptophan decarboxylase tryptophan metabolic pathways have been identified in certain members of the human gut microbiota, such as *Clostridium*, which is able to decarboxylate tryptophan, leading to the production of the neurotransmitter tryptamine (Williams et al., 2014). 16 S rDNA sequencing in our previous study (Xuan-Qing et al., 2021) also showed that the intestinal flora of mice in the DSS group was disordered compared to the control group. After treatment with BTW, the diversity of intestinal flora was significantly improved. At the phylum level, the proportion of Firmicutes to Bacteroidetes was decreased, and the ratio of Proteobacteria was decreased. At the genus level, the relative abundance of Escherichia-Shigella was decreased, but that of *Lactobacillus* and Akkermansia were increased. Therefore, the role of gut flora in the regulation of Trp metabolism by BTW still needs to be further experimental validation.

## 5 Conclusion

In conclusion, the study is an exploratory study, we proposed a new mechanism for the treatment of UC in mice with BTW. BTW promotes the expression of colonic DDC. Indole metabolites, such as I3A and IET, are increased and kynurenine is decreased in the colon of the BTW group. BTW regulates Trp metabolism from the kynurenine pathway to the indole metabolism pathway. BTW regulated Trp metabolism to increase metabolite levels, activate the AHR/IL-22 pathway to restore intestinal barrier function, and significantly alleviated DSS-induced colitis. These results confirmed the role of DDC in Trp metabolism and UC, enrich the understanding of the therapeutic effect of BTW on UC, and provided a theoretical basis for the clinical application of BTW.

## Data Availability

The original contributions presented in the study are included in the article/Supplementary Material, further inquiries can be directed to the corresponding authors.

## Abbreviations

AB/PAS, Alcian Blue/Phosphate Acid Schiff; AHR, Aryl hydrocarbon receptor; BTW, Baitouweng decoction; DAI, disease activity index; DDC, dopa decarboxylase; DSS, dextran sodium sulfate; H&E, hematoxylin-eosin; I3A, indole-3-acetic acid; IET, tryptophol; IFN-γ, interferon gamma; IL-6, ofinterleukin-6; Kyn, kynurenine; LC-MS/MS, liquid chromatography-tandem mass spectrometry; TJ, tight junction; Trp, L-tryptophan; PCA, Principal Component Analysis; PCC, Pearson’s Correlation Coefficient; qPCR, real-time quantitative PCR; SCFAs, short-chain fatty acid; TMP, Tryptophan metabolism; UC, Ulcerative colitis; SASP, Sulfasalazine; UHPLC, ultra-high performance liquid chromatography; UPLC, ultrahigh performance liquid chromatography.
