# Supplementary figures and images for: Baitouweng decoction alleviates ulcerative colitis by regulating tryptophan metabolism through DOPA decarboxylase promotion
Source: Front Pharmacol. 2024 Jun 21;15:1423307. doi: 10.3389/fphar.2024.1423307 (PMC11224817; doi:10.3389/fphar.2024.1423307)

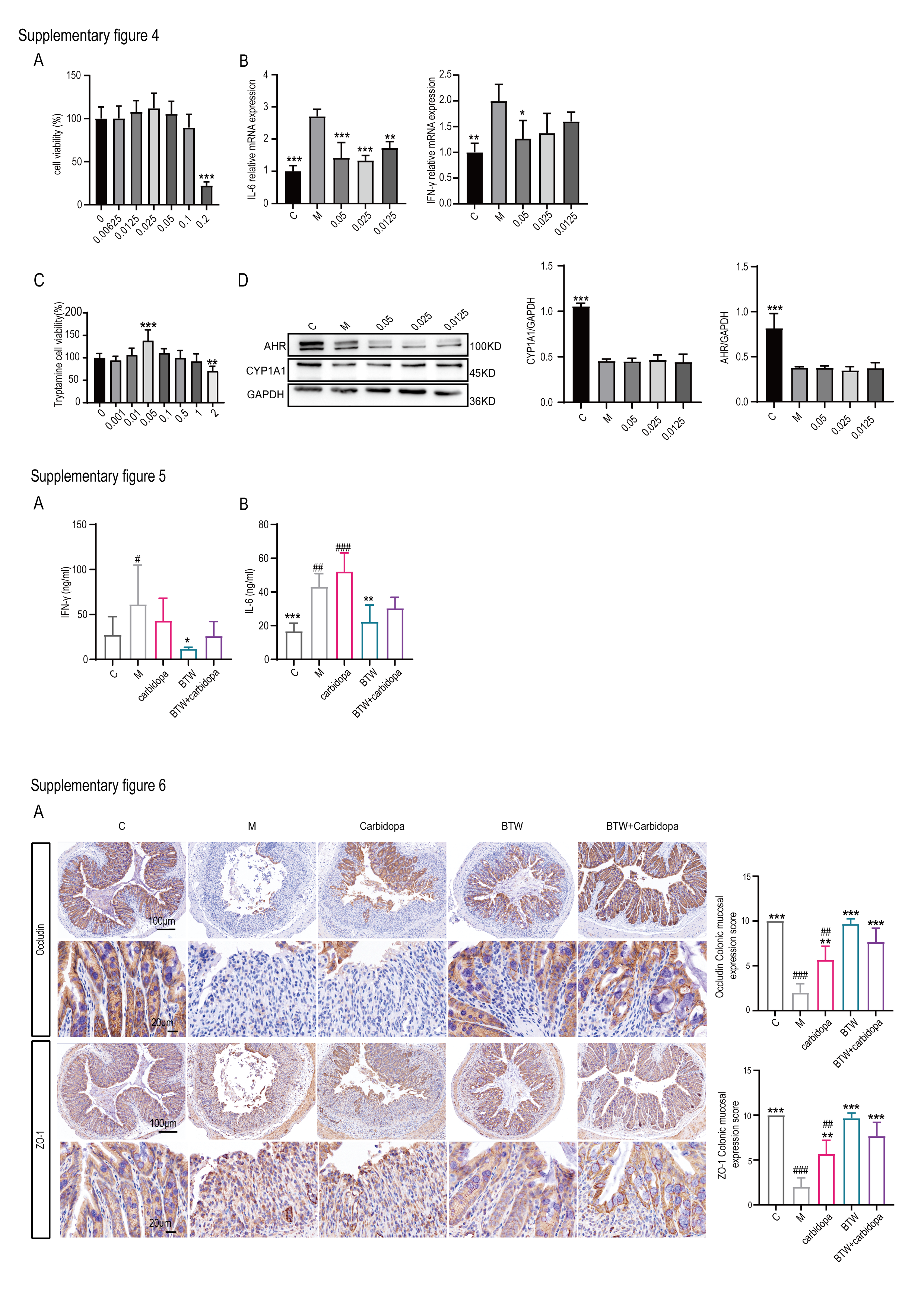

Supplement: Supplementary file 1 [file Image2.PNG]

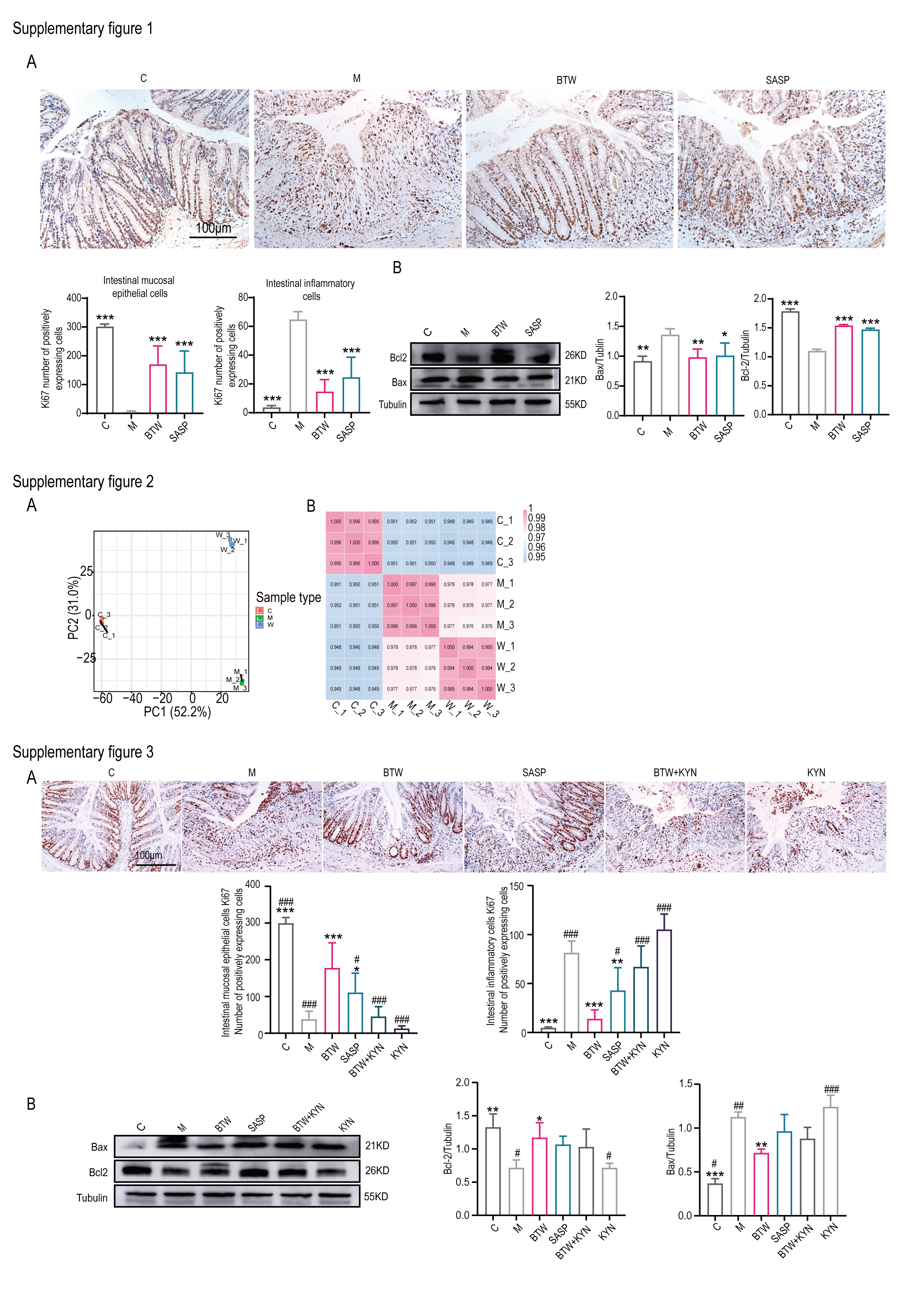

Supplement: Supplementary file 2 [file Image1.PNG]
